# Radiosensitization by the histone deacetylase inhibitor vorinostat under hypoxia and with capecitabine in experimental colorectal carcinoma

**DOI:** 10.1186/1748-717X-7-165

**Published:** 2012-09-27

**Authors:** Marie Grøn Saelen, Anne Hansen Ree, Alexandr Kristian, Karianne Giller Fleten, Torbjørn Furre, Helga Helseth Hektoen, Kjersti Flatmark

**Affiliations:** 1 Department of Tumor Biology, Norwegian Radium Hospital, Oslo University Hospital, P.O. Box 4953, Nydalen, 0424, Oslo, Norway; 2 Institute of Clinical Medicine, University of Oslo, Oslo, Norway; 3 Department of Oncology, Akershus University Hospital, Lørenskog, Norway; 4 Department of Medical Physics, Norwegian Radium Hospital, Oslo University Hospital, Oslo, Norway

**Keywords:** Rectal cancer, Vorinostat, Fluoropyrimidine, Hypoxia, Radiation

## Abstract

**Background:**

The histone deacetylase inhibitor vorinostat is a candidate radiosensitizer in locally advanced rectal cancer (LARC). Radiosensitivity is critically influenced by hypoxia; hence, it is important to evaluate the efficacy of potential radiosensitizers under variable tissue oxygenation. Since fluoropyrimidine-based chemoradiotherapy (CRT) is the only clinically validated regimen in LARC, efficacy in combination with this established regimen should be assessed in preclinical models before a candidate drug enters clinical trials.

**Methods:**

Radiosensitization by vorinostat under hypoxia was studied in four colorectal carcinoma cell lines and in one colorectal carcinoma xenograft model by analysis of clonogenic survival and tumor growth delay, respectively. Radiosensitizing effects of vorinostat in combination with capecitabine were assessed by evaluation of tumor growth delay in two colorectal carcinoma xenografts models.

**Results:**

Under hypoxia, radiosensitization by vorinostat was demonstrated *in vitro* in terms of decreased clonogenicity and *in vivo* as inhibition of tumor growth. Adding vorinostat to capecitabine-based CRT increased radiosensitivity of xenografts in terms of inhibited tumor growth.

**Conclusions:**

Vorinostat sensitized colorectal carcinoma cells to radiation under hypoxia *in vitro* and *in vivo* and improved therapeutic efficacy in combination with capecitabine-based CRT *in vivo*. The results encourage implementation of vorinostat into CRT in LARC trials.

## Background

In locally advanced rectal cancer (LARC), neoadjuvant chemoradiotherapy (CRT) is given to obtain tumor down-staging to allow complete surgical removal, and single-agent fluoropyrimidine in combination with fractionated pelvic radiation remains the standard regimen [1]. Treatment responses vary considerably, and this may be particularly important in large T4 tumors that depend greatly on the effect of neoadjuvant CRT for preoperative down-staging [2]. Other potential radiosensitizing agents have been evaluated for their ability to further enhance local tumor response, but improvement has so far not been achieved, warranting the continued search for novel radiosensitizers [3-6]. Histone deacetylase (HDAC) inhibitors have emerged as a new class of drugs that has been shown to sensitize tumors to radiation in experimental models. We have previously assessed the radiosensitizing ability of the HDAC inhibitor vorinostat in experimental colorectal carcinoma models, demonstrating reduced *in vitro* clonogenicity upon radiation exposure and delayed tumor growth of xenografts exposed to fractionated radiation [7]. In a recent clinical phase I study, we reported a favorable toxicity profile of vorinostat in combination with pelvic palliative radiotherapy [8,9].

As recently highlighted in guidelines from the NCRI Clinical and Translational Radiotherapy Research Working Group [10], novel radiosensitizers must be adequately evaluated in relevant preclinical models in order to justify exposing patients to the risks of adding a new drug to radiotherapy or CRT. Since human solid tumors, including rectal carcinomas, often contain a substantial fraction of hypoxic cells that are intrinsically more resistant to radiotherapy, a drug’s ability to radiosensitize tumor cells under hypoxia should be taken into account when investigating new CRT candidates [11]. Furthermore, since fluoropyrimidine-based CRT is the established regimen in LARC, potential interaction between a new drug and the standard treatment should be investigated to reveal possible antagonistic or synergistic effects. In the present work, radiosensitizing effects of vorinostat were assessed under hypoxic conditions in four colorectal carcinoma models *in vitro* and in one xenograft model. Moreover, radiosensitizing properties of vorinostat in combination with the fluoropyrimidine capecitabine were investigated in two colorectal carcinoma xenograft models.

## Methods

### Experimental treatments

Ionizing radiation (IR) was delivered to cell lines in culture at a rate of 1.0 Gy/min by Faxitron Cabinet X-ray system (model 43855 F with CP 160 Option; Faxitron Bioptics, Lincolnshire, IL). Control cells were simultaneously placed in room temperature. To tumor xenografts, IR was delivered in daily 2-Gy fractions using a 6-MV photon beam from a linear accelerator (Varian Clinac 2100 CD; Varian, San Diego, CA), at a dose rate of 2.6 Gy/min. Control mice were anaesthetized and brought to the radiation room. Vorinostat (Alexis Biochemicals, Lausen, Switzerland) and capecitabine (Roche, Basel, Switzerland) were prepared and stored as previously described [7].

In experiments involving hypoxia, the following single agent and combination treatments were given: C (control) = NO (normoxia), HO (hypoxia), IR-NO (IR under normoxia), IR-HO (IR under hypoxia), VOR-NO (vorinostat under normoxia), VOR-HO (vorinostat under hypoxia), VOR-IR-NO (vorinostat and IR under normoxia), and VOR-IR-HO (vorinostat and IR under hypoxia). For experiments involving combination of vorinostat and capecitabine, the following treatments were given: C (control), VOR (vorinostat), CAP (capecitabine), IR, VOR-IR (vorinostat and IR), CAP-IR (capecitabine and IR), and VOR-CAP-IR (vorinostat, capecitabine, and IR).

### Cell lines and in vitro experiments

Human colorectal carcinoma cell lines HCT116, HT29, and SW620 (ATCC, Manassas, VA) and KM20L2 (kindly provided by Dr. M. R. Boyd, National Cancer Institute, Frederick, MD) were used. The cell lines were free from mycoplasma infection and cell line identity was validated by short tandem repeat analysis. Culturing conditions were previously described [7]. Vorinostat (1 or 2 μM) was added to the cell cultures for an incubation period of 18 h. *In vitro* hypoxia (1% O_2_) was generated using an Invivo2 200 Hypoxic Workstation (Ruskinn, Bridgend, UK). Cell cultures were incubated under these conditions for 18 h before sealing the flasks using non-filter caps and transferring them to an x-ray unit. After IR exposure, culture flasks were transferred to normoxic conditions. Control cell cultures were kept under normoxic conditions at all times. Clonogenicity was performed as previously described [7] with the main modification being that cells were grown in T-25 flasks (Nunc, Roskilde, Denmark) to allow generation of hypoxia. Plating efficiencies determined from control experiments were 0.63 ± 0.07 for HCT116, 0.84 ± 0.19 for HT29, 0.61 ± 0.14 for SW620, and 0.78 ± 0.08 for KM20L2. Surviving fractions (SF) were calculated relative to the relevant control.

For analysis of HIF-1α induction, HCT116 cells were seeded in cell culture flasks and exposed to HO or NO as previously described or treated with 100 μM CoCl_2_ for 4 h to generate a positive control for HIF-1α expression (Sigma Aldrich, St Louis, MO). Whole cells lysates were generated as previously described [12] and stored at - 80°C until analysis. Separation of 7.5 μg of protein was performed using 4-12% NuPAGE® Novex Bis-Tris Gels (Invitrogen, Carlsbad, CA) in MES buffer and transferred to Immobilon-P membranes (Millipore, Bedford, MA). Membranes were blocked for 1 h at room temperature in Tris-buffered saline with 0.1% Tween-20 (TBST) and 5% non fat dry milk and incubated over night at 4°C with mouse anti-HIF-1α antibody (# 610958; BD Transduction Laboratories, Franklin Lakes, NJ) or goat anti-actin antibody (sc-1616; Santa Cruz Biotechnology, Santa Cruz, CA). After washing, the membranes were incubated for 1 h at room temperature with appropriate horseradish peroxidase conjugated secondary antibody, and bands were visualized using Super Signal West Dura Extended Duration Substrate (Thermo Scientific, Waltham, MA).

### Animal models

Locally bred female and male athymic Balb/c mice 6–8 weeks old were used. For one experiment (vorinostat, capecitabine, and IR– in vivo tumor growth, HCT116 xenografts) Balb/c nude (nu/nu) mice from Harlan Laboratories (Rossdorf, Germany) were purchased, as our animal facility had reduced availability of inbred mice due to relocation of the department. The mice were maintained under specific pathogen-free conditions, and food and water were supplied *ad libitum*. Housing and all procedures involving animals were performed according to protocols approved by the Animal Care and Use Committee, in compliance with the National Committee for Animal Experiment’s guidelines on animal welfare. Xenografts were established as previously described [7] on the thigh (hypoxia experiments) or on the rear flank (capecitabine experiments) and tumor volumes were calculated using the following formula: volume = (п/6) × a × b^2^, in which a and b were the largest and the smallest perpendicular tumor diameters, respectively. The mice were sacrificed when tumors reached a diameter of 15 mm (thigh) or 20 mm (flank), or if the animal failed to thrive.

### In vivo experiments

*In vivo* tumor hypoxia was achieved by placing a heavy clamp over the proximal thigh of anesthetized mice during irradiation [13]. The tumors were clamped for 3.5 min before and during IR exposure (to a total period of 5.0 min). In experiments involving hypoxia, the mice were randomized by tumor volume into groups of 5–6 animals and were treated with vorinostat concomitantly to irradiation for four consecutive days. Vorinostat (100 mg/kg) or vehicle was given daily by intraperitoneal (i.p.) injections three hours before radiation. Tumor blood supply to clamped xenografts was examined by measuring tumor radioactivity after injection of I^125^ and used as a measure of acute hypoxia. I^125^ (Hartmann Analytic, Braunschweig, Germany) in the form of an iodinated antibody and with an activity of 2.85 MBq/ml, was administered by tail vein injection (100 μL, mean activity of 10 kBq/g) to four anesthetized mice (tumor volume 91.3 ± 34.3 mm^3^; mean ± standard deviation). After 5 min, the mice were sacrificed and tumors were dissected and clamped and unclamped tumors were analyzed by a gamma counter (COBRA II Auto-Gamma; Packard Canberra, Meriden, CT). The ratio of I^125^ activity (*i.e.,* activity in clamped tumor divided by activity in unclamped tumor on the same mouse) was 0.03 ± 0.01 (mean ± standard deviation), indicating a substantial reduction of blood flow to clamped tumors (p < 0.001).

In experiments involving capecitabine, the mice were randomized by tumor volume into treatment groups of 6–9 mice and treated with vorinostat concomitantly to irradiation for five consecutive days. Vorinostat (100 mg/kg) or vehicle was given daily by i.p. injections three hours before radiation. Capecitabine (359 mg/kg) or vehicle was given daily by oral gavage immediately after administration of vorinostat. Relative tumor volumes (RTV) were calculated relative to the tumor volume on the day of treatment initiation. For each tumor, tumor doubling time (T_2x_) relative to the Day 1 tumor volume was determined. Tumor growth delay (TGD_2x_) was calculated by subtracting the mean T_2x_ of the vehicle-treated tumors from the T_2x_ for each treated xenograft.

### Statistical analysis

Statistical analysis was performed using SPSS 16.0 (SPSS Inc., Chicago, IL). Differences between groups were analyzed using the two-sided Student *t* test under conditions of normality and a non-parametric test (Mann–Whitney rank-sum test) under other conditions. *p* values less than 0.05 were considered statistically significant.

## Results

### Vorinostat, hypoxia, and IR – in vitro clonogenicity

**Figure 1:**
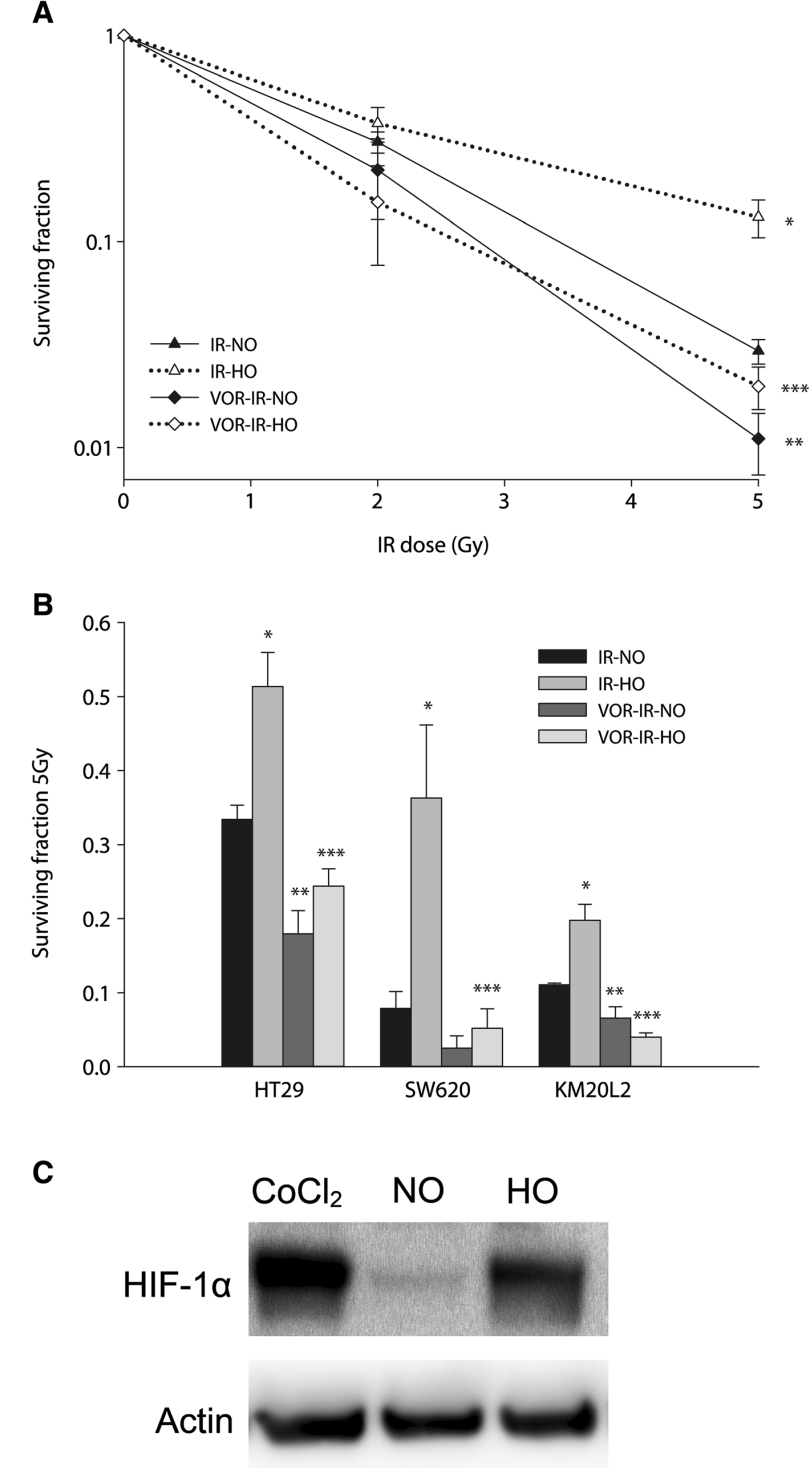
**Vorinostat, hypoxia, and ionizing radiation (IR) – *in vitro *clonogenicity and HIF-1α expression.** (**A**) HCT116, (**B**) HT29, KM20L2 and SW620 cells were treated with IR under normoxia (IR-NO), IR under hypoxia (IR-HO), vorinostat and IR under normoxia (VOR-IR-NO) or vorinostat and IR under hypoxia (VOR-IR-HO). Surviving fractions are shown as mean, and bars represent SEM. * Indicates significant difference between IR-NO and IR-HO. ** Indicates significant difference between VOR-IR-NO and IR-NO. *** Indicates significant difference between VOR-IR-HO and IR-HO. (**C**) Western immunoblot analysis of HIF-1α expression in HCT116 cells exposed to normoxia (NO) or hypoxia (HO). Cells were treated with CoCl_2_ as a positive control and actin was used as protein loading control.

In initial experiments on HCT116 cells, clonogenicity was not influenced by hypoxia (SF for HO cells was 0.97 ± 0.08; mean ± standard error of the mean, compared to C). Likewise, the survival of VOR-NO cells and VOR-HO cells was similar (SF were 0.35 ± 0.04 and 0.41 ± 0.05, respectively). The presence of hypoxia was verified by western immunoblot analysis, showing induction of HIF-1α after exposure to *in vitro* hypoxia. In contrast, IR-HO cells were less sensitive to a radiation dose of 5 Gy than IR-NO cells (SF were 0.13 ± 0.03 and 0.029 ± 0.004, respectively; p = 0.002). Notably, vorinostat enhanced the 5-Gy radiation effects of both hypoxic and normoxic cells (SF for VOR-IR-HO cells was 0.020 ± 0.005; p = 0.003; SF for VOR-IR-NO cells was 0.011 ± 0.004; p = 0.006). The experiments were also performed applying 2-Gy radiation doses, and although the effects of hypoxia and vorinostat treatment exhibited the same trends as for the 5-Gy dose, the differences were not statistically significant (Table 1, Figure 1).

**Table 1:** ***In vitro *clonogenicity - mean surviving fractions (SEM) after treatment with ionizing radiation (IR, 5 Gy), vorinostat and hypoxia**

	**HCT116**		**HT29**		**SW620**		**KM20L2**	
	***SF***	***p***	***SF***	***p***	***SF***	***p***	***SF***	***p***
*Monotherapy (relative to control)*								
IR	0.029 (0.04)	<0.001	0.33 (0.02)	<0.001	0.079 (0.02)	<0.001	0.11 (0.002)	<0.001
HO	0.97 (0.08)	0.7	0.98 (0.03)	0.6	0.62 (0.28)	0.3	0.92 (0.01)	0.01
VOR	0.35 (0.04)	<0.001	0.78 (0.07)	0.05	0.84 (0.04)	0.07	0.66 (0.04)	0.04
*Combination therapy*								
IR-HO (relative to HO)	0.13 (0.03)	<0.001	0.51 (0.05)	0.002	0.36 (0.10)	0.02	0.20 (0.02)	<0.001
VOR-HO (relative to HO)	0.41 (0.05)	<0.001	0.88 (0.09)	0.3	0.68 (0.14)	0.1	0.68 (0.03)	0.08
VOR-IR (relative to VOR)	0.011 (0.004)	<0.001	0.18 (0.03)	<0.001	0.026 (0.02)	<0.001	0.066 (0.02)	<0.001
VOR-IR-HO (relative to VOR-HO)	0.020 (0.005)	<0.001	0.24 (0.02)	<0.001	0.052 (0.03)	<0.001	0.040 (0.006)	<0.001

SF = surviving fractions; HO = hypoxia; VOR = vorinostat; IR-HO = IR under hypoxia;

VOR-HO = vorinostat under hypoxia; VOR-IR = vorinostat and IR; VOR-IR-HO = vorinostat and IR under hypoxia.

In subsequent experiments, a radiation dose of 5 Gy was used. Hypoxia alone did not substantially influence clonogenicity in any of the models. The cytotoxic effect of vorinostat alone varied among the cell lines (SF 0.66-0.88), but was not significantly different for VOR-HO cells compared to VOR-NO cells. Clonogenicity of IR-HO cells was higher than for the respective IR-NO counterparts (for HT29, SF 0.51 ± 0.05 *versus* 0.33 ± 0.02; p = 0.02; for SW620, SF 0.36 ± 0.10 *versus* 0.079 ± 0.02; p = 0.049; for KM20L2, SF 0.20 ± 0.02 *versus* 0.11 ± 0.002; p = 0.02). Again, in VOR-IR-HO cells compared to IR-HO cells, vorinostat caused radiosensitization (for HT29, SF 0.24 ± 0.02 *versus* 0.51 ± 0.05; p = 0.005; for SW620, SF 0.052 ± 0.03 *versus* 0.36 ± 0.10; p = 0.04; for KM20L2, SF 0.040 ± 0.006 *versus* 0.20 ± 0.02; p = 0.002). Under normoxia (VOR-IR-NO), HT29 and KM20L2 were radiosensitized by vorinostat, while the effect was not statistically significant for SW620.

### Vorinostat, hypoxia, and IR – in vivo tumor growth

**Figure 2:**
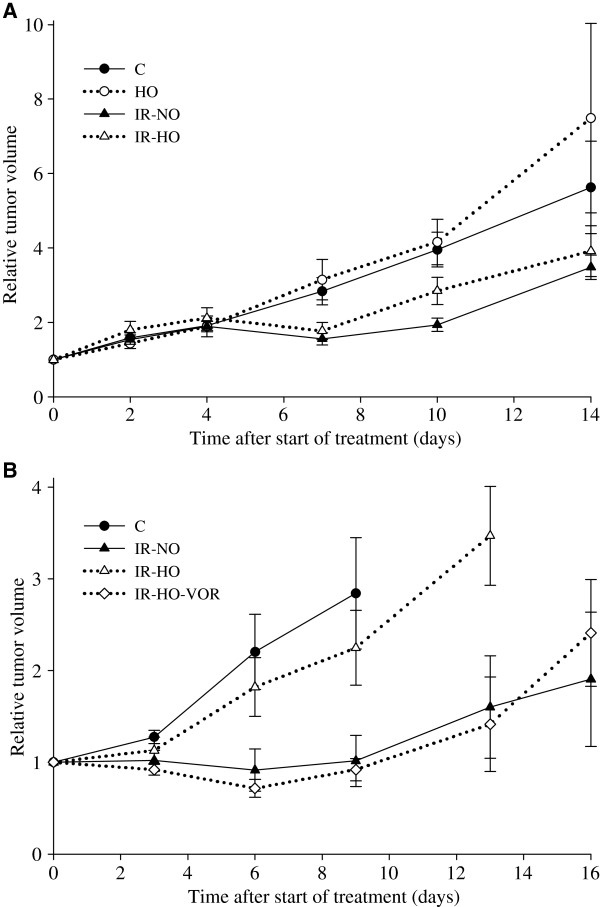
**Vorinostat, hypoxia, and ionizing radiation (IR) – *in vivo *tumor growth.** (**A**) Mice bearing SW620 xenografts were treated with vehicle (control, C), hypoxia (HO), IR under normoxia (IR-NO) and IR under hypoxia (IR-HO). (**B**) Mice bearing SW620 xenografts were treated with vehicle (control, C), IR under normoxia (IR-NO), IR under hypoxia (IR-HO), or vorinostat and IR under hypoxia (VOR-IR-HO). Relative tumor volumes (mean ± SEM) presented as function of time after start of treatments.

In a pilot experiment, radiation exposure inhibited growth of normoxic SW620 xenografts but not of hypoxic tumors. 5–6 mice were randomized to each treatment group (control, C; hypoxia, HO: ionizing radiation under normoxia, IR-NO; ionizing radiation under hypoxia, IR-HO). Experimental treatments were started 17 days after establishment of SW620 xenografts at tumor volumes of 192.7 ± 112 mm^3^ (mean ± standard deviation). Of 23 mice included in the experiment, one IR-NO animal was excluded from analysis as it was sacrificed early (day 4) due to anesthesia-related complications. Untreated xenografts had a T_2x_ of 5.33 ± 2.6 days. The TGD_2x_ of HO tumors was unchanged compared to C (TGD_2x_ 0.27 ± 1.9 days; p = 0.74). The IR exposure inhibited growth of normoxic xenografts as IR-NO tumors were growth inhibited (TGD2x 5.47 ± 1.9 days; p < 0.001), while the growth rate of IR-HO tumors was not significantly different from C (TGD_2x_ 2.33 ± 3.0; p = 0.09) (Table 2, Figure 2).

**Table 2:** Vorinostat, hypoxia, and ionizing radiation - tumor growth delay

**Treatment groups**	**TGD**_**2x**_**(days)**	**Compared to (group)**	**p-value**
**SW620 Pilot**			
HO	0.27 ± 1.9	C	0.7
IR-NO	5.47 ± 1.9	C	<0.001
IR-HO	2.33 ± 3.0	C	0.09
**SW620 Main experiment**			
IR-NO	7.83 ± 5.9	C	0.02
IR-HO	−1.43 ± 3.6	C	0.4
VOR-IR-HO	6.07 ± 2.5	IR-HO	0.02

TGD_2x_ = tumor growth delay at 2-fold increase of relative tumor volume; C = control; HO = hypoxia; IR-NO = IR under normoxia; IR-HO = IR under hypoxia; VOR-IR-HO = vorinostat and IR under hypoxia.

A subsequent experiment was undertaken to specifically investigate the effects of vorinostat under hypoxia, involving four treatment groups: C, IR-NO, IR-HO, or VOR-IR-HO. Experimental treatments were started 13 days after establishment of SW620 xenografts at tumor volumes of 51.1 ± 35 mm^3^ (mean ± standard deviation). Of 23 mice included in the experiment, four mice were excluded from analysis as they were sacrificed early (days 1–2) for the following reasons: anesthesia-related complications (*n* = 3; two VOR-IR-HO and one IR-NO) and failure to thrive (*n* = 1; VOR-IR-HO), leaving three animals for analysis in the VOR-IR-HO group. For untreated xenografts T_2x_ was 9.60 ± 7.7 days. Similarly to the pilot findings, radiation exposure inhibited growth under normoxia but not under hypoxia (for IR-NO TGD_2x_ was 7.83 ± 5.9 days; p = 0.016 compared to C); for IR-HO tumors TGD_2x_ was −1.43 ± 3.6; p = 0.40 compared to C)). Importantly, vorinostat enhanced radiosensitivity under hypoxia as growth of the VOR-IR-HO xenografts was inhibited compared to IR-HO xenografts (TGD_2x_ was 6.07 ± 2.5 versus −1.43 ± 3.6; p = 0.015).

### Vorinostat, capecitabine, and IR – in vivo tumor growth

**Figure 3:**
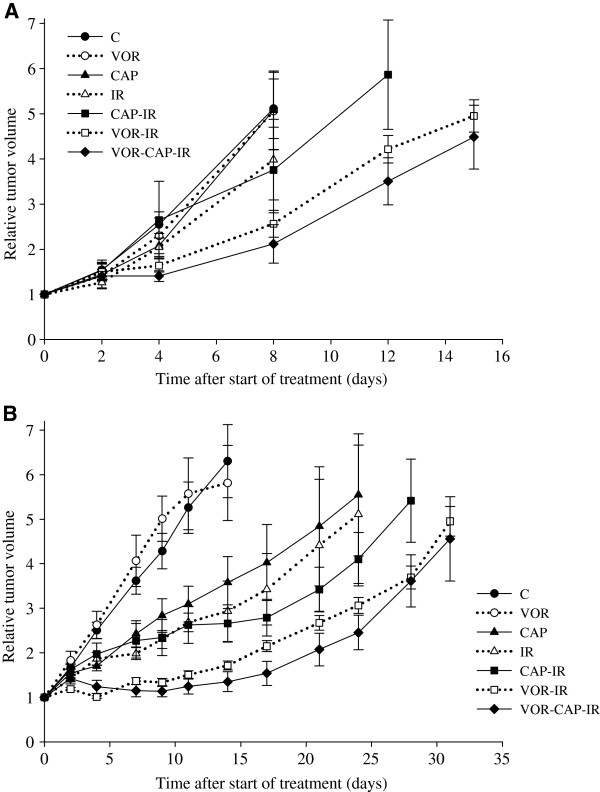
**Vorinostat, capecitabine, and ionizing radiation (IR) – *in vivo *tumor growth.** Mice bearing (**A**) SW620 and (**B**) HCT116 xenografts were treated with vehicle (control, C), vorinostat (VOR), capecitabine (CAP), IR, capecitabine and IR (CAP-IR), vorinostat and IR (VOR-IR), or vorinostat, capecitabine and IR (VOR-CAP-IR). Relative tumor volumes (mean ± SEM) presented as function of time after start of treatments.

To assess the effect of adding vorinostat to fluoropyrimidine-based CRT, two xenograft models, SW620 and HCT116, were used. The experimental setup included seven groups of mice receiving C, VOR, CAP, IR, VOR-IR, CAP-IR, or the full combination of VOR-CAP-IR. Experimental treatments were initiated 10 days (SW620) or 14 days (HCT116) after establishment of xenografts at tumor volumes of 100.8 ± 86 mm^3^ and 77.0 ± 57 mm^3^ (mean ± standard deviation), respectively. Of 53 mice included in each experiment, five mice were sacrificed early (days 1–4) because of anesthesia-related complications (*n* = 3; SW620) or failure to thrive (*n* = 2; HCT116), and were excluded from analysis. Furthermore, due to ulceration of the tumors, three xenografts did not reach T_2x_ and one vehicle xenograft grew abnormally (HCT116) and were removed from the study (Table 3, Figure 3).

**Table 3:** Vorinostat, capecitabine, and ionizing radiation - tumor growth delay

**Treatment groups**	**TGD**_**2x**_**(days)**	**Compared to (group)**	**p-value**
**HCT116**			
IR	5.68 ± 8.5	C	0.03
CAP	2.68 ± 2.4	C	0.001
VOR	0.24 ±1.9	C	0.7
CAP-IR	5.46 ± 6.1	IR	0.9
VOR-IR	20.06 ± 15.1	IR	0.01
VOR-CAP-IR	18.99 ± 7.3	CAP-IR	<0.001
**SW620**			
IR	1.40 ± 2.2	C	0.06
CAP	0.14 ± 1.8	C	0.8
VOR	0.88 ± 1.9	C	0.2
CAP-IR	2.53 ± 3.1	IR	0.3
VOR-IR	2.8 ± 2.6	IR	0.2
VOR-CAP-IR	5.78 ± 3.9	CAP-IR	0.03

TGD_2x_ = tumor growth delay at 2-fold increase of relative tumor volume; IR = ionizing radiation;

C = control; CAP = capecitabine; VOR = vorinostat; CAP-IR = capecitabine and IR;

VOR-IR = vorinostat and IR; VOR-CAP-IR = vorinostat, capecitabine and IR.

For untreated xenografts T_2x_ was 4.13 ± 0.9 days for HCT116 and 4.30 ± 1.3 days for SW620. While IR inhibited growth of HCT116 (TGD_2x_ was 5.68 ± 8.5 days; p = 0.025 compared to C), growth inhibition of SW620 was not statistically significant (TGD_2x_ was 1.40 ± 2.2 days, p = 0.061 compared to C). Single-agent therapy with CAP delayed tumor growth compared to C for HCT116 (TGD_2x_ was 2.68 ± 2.4 days, p = 0.001), but not for SW620 (TGD_2x_ was 0.14 ± 1.8 days, p = 0.8). Single-agent therapy with VOR did not alter tumor growth (for HCT116, TGD_2x_ was 0.24 ± 1.9 days, p = 0.7 compared to C, respectively; for SW620, TGD_2x_ was 0.88 ± 1.9 days, p = 0.2 compared to C. Tumor growth delay of VOR-IR xenografts was observed for HCT-116 (TGD_2x_ of VOR-IR was 20.06 ± 15.1 days compared to TGD_2x_ of IR which was 5.68 ± 8.5 days, p = 0.01), while for SW620 tumor growth was not significantly delayed, although a similar trend was observed (TGD_2x_ of VOR-IR was 2.78 ± 2.6 days compared to TGD_2x_ of IR which was 1.40 ± 2.2 days, p = 0.2). Only minor, non-significant changes of radiosensitivity was obtained by adding capecitabine to IR (CAP-IR) (for HCT116, TGD_2x_ of CAP-IR was 5.46 ± 6.1 days compared to TGD_2x_ of IR of 5.68 ± 8.5 days, p = 0.9; for SW620, TGD_2x_ of CAP-IR was 2.53 ± 3.1 days compared to TGD_2x_ of IR of 1.40 ± 2.2 days, p = 0.2). Notably, vorinostat in combination with capecitabine improved radiation efficacy in both models as TGD_2x_ of VOR-CAP-IR xenografts were significantly increased compared to TGD_2x_ of CAP-IR xenografts (for HCT116, TGD_2x_ of VOR-CAP-IR was 18.99 ± 7.3 days compared to TGD_2x_ of 5.46 ± 6.1 days for CAP-IR, p <0.001; for SW620, TGD_2x_ of VOR-CAP-IR was 5.78 ± 3.9 days compared to TGD_2x_ of 2.53 ± 3.1 days for CAP-IR, p = 0.03).

## Discussion

Exposure to hypoxic conditions during radiation made the cells in our *in vitro* models more radioresistent, in line with theories of classical radiobiology stating that hypoxia increases the resistance of cancer cells to radiation treatment. *In vivo*, a similar effect of short-term acute hypoxia was observed, as clamped tumors were more resistant to treatment with fractionated radiation than unclamped tumors. Vorinostat enhanced radiosensitivity of cells exposed to hypoxia during radiation in all colorectal carcinoma cell lines *in vitro*, almost counter-balancing hypoxia-induced radioresistance. In line with these results*,* vorinostat demonstrated radiosensitizing effects in xenografts exposed to acute hypoxia, as tumor volumes of vorinostat-treated mice irradiated under hypoxia were similar to the tumor volumes of mice irradiated under normoxia, reversing the radioresistent hypoxic phenotype.

Response to cancer treatment is strongly influenced by the tumor microenvironment, and specifically, the most important determinant of radiotherapy response is tissue oxygenation. Colorectal tumors are often large, and analysis of hypoxia biomarkers in patient samples has revealed that most tumors exhibit a molecular phenotype consistent with varying hypoxia [11]. Moreover, in a recent report, expression of the hypoxia-associated protein carbonic anhydrase IX was negatively associated with CRT response in patients with rectal cancer [14]. Hence, a likely explanation for variable CRT response in rectal cancer is variable tumor oxygenation, and in this perspective, the ability to overcome hypoxia-related radioresistance would be an advantageous property of a radiosensitizing drug. In other tumor forms, a small number of drugs (such as gemcitabine, irinotecan, and the poly(ADP-ribose) polymerase-inhibitor velaparib) [15-17] have been evaluated in combination with radiotherapy under hypoxic conditions. Considering the importance of tissue oxygenation for radiotherapy efficacy, the scarcity of experimental data exploring radiosensitizers under hypoxia in LARC is remarkable. One reason for this could be related to difficulties in selecting appropriate models for mimicking clinical hypoxia when evaluating radiosensitizing drugs. This challenge was also apparent in our experiments, as the impact of hypoxia on radioresistance was less pronounced in the pilot than in the main experiment (Figure 2). This illustrates biological variation which may be partially explained by the introduction of a tighter set of hypoxia clamps in the second experimental series than in the pilot. There is also some controversy regarding how to conduct *in vivo* experiments, particularly when fractionating the radiation. Although the clamping technique has been shown to be relevant for this purpose [18], it has been questioned whether this strategy truly reflects radiosensitivity under hypoxia [19]. In this context, our approach of administering vorinostat prior to short-term hypoxia and irradiation is interesting, as our experiments represent an important attempt to evaluate a novel radiosensitizer under tumor hypoxia. However, such experiments should be repeated in larger series of animals, and with the paucity of data describing the use of conventional radiosensitizing agents in LARC under hypoxia, the therapeutic implication of vorinostat in this clinical setting is still elusive.

In LARC, fluoropyrimidine-based neoadjuvant CRT is the established treatment regimen and any novel radiosensitizing agent must be evaluated in this context. In the present study, *in vivo* addition of vorinostat to capecitabine and fractionated radiation enhanced treatment efficacy in terms of inhibited tumor growth in two colorectal xenograft models. Vorinostat has recently been shown to synergize with fluoropyrimidine-based chemotherapy in preclinical colorectal carcinoma models [20,21], but radiosensitizing properties of the combination has to our knowledge not been evaluated. Applying radiation in combination with vorinostat or capecitabine alone, we observed trends towards enhanced radiosensitivity, although the reduction of tumor volume was not statistically significant. In our previous experiments, both these drugs were potent radiosensitizers in experimental colorectal carcinoma models [7,22]. The variable effects on radiosensitivity could be explained by the limited total treatment doses possible to administer in the experimental setting, in addition to variation associated with the biological complexity of animal models. Similar limitations were also present in the combination group and in this context, the enhanced radiosensitivity observed when combining vorinostat and capecitabine is noteworthy, and adds to the preclinical evidence supporting vorinostat as radiosensitizer in LARC [7,23].

## Conclusion

Although preclinical evidence [7,23] and clinical safety data [8,9] both support the use of vorinostat as radiosensitizer in LARC, more extensive preclinical examination was necessary prior to recommending vorinostat as an additional component of CRT in LARC trials. Hence, we expanded our *in vitro* and *in vivo* models by studying radiosensitizing effects of vorinostat under hypoxic conditions and in combination with capecitabine. Importantly, the results from the present study indicate that vorinostat is a radiosensitizer under hypoxic conditions and also interacts favorably with capecitabine in experimental models of colorectal carcinoma. Recognizing the important role of hypoxia in LARC, particularly in large T4 tumors, as well as the requirement that novel drugs should be compatible with fluoropyrimidine-based standard CRT, our results encourage the implementation of vorinostat in next-generation clinical CRT trials in LARC.

## Abbreviations

LARC: Locally advanced rectal cancer; CRT: Chemoradiotherapy; HDAC: Histone deacetylase; IR: Ionizing radiation; C: Control; HO: Hypoxia; IR-NO: IR under normoxia; IR-HO: IR under hypoxia; VOR: Vorinostat; VOR-IR: Vorinostat and IR; VOR-NO: Vorinostat under normoxia; VOR-HO: Vorinostat under hypoxia; VOR-IR-NO: Vorinostat and IR under normoxia; VOR-IR-HO: Vorinostat and IR under hypoxia; CAP: Capecitabine; CAP-IR: Capecitabine and IR; VOR-CAP-IR: Vorinostat, capecitabine, and IR; SF: Surviving fractions; i.p: Intraperitoneal; T_2x_: Tumor doubling time; TGD: Tumor growth delay.

## Competing interests

The authors declare that they have no competing interests.

## Authors’ contributions

MGS participated in study design, carried out the *in vitro* and *in vivo* radiation experiments, and drafted the manuscript. AHR and KF participated in study design, data interpretation and in writing the manuscript. TF helped with technical assistance on the *in vivo* radiation. AK and KGF assisted with the *in vivo* radiation experiments. HHH carried out the western blot analysis. All authors read and approved the final manuscript.
